# Interactions between anti-EGFR therapies and cytotoxic chemotherapy in oesophageal squamous cell carcinoma: why clinical trials might have failed and how they could succeed

**DOI:** 10.1007/s00280-020-04187-w

**Published:** 2020-11-09

**Authors:** Madusha Meemanage, Lindsay C. Spender, Diane Collinson, Joanna Iannetta, Pranavi Challapalli, Julie Turbitt, Caroline Clark, Mark Baxter, Graeme Murray, Shaun Walsh, Zofia Miedzybrodzka, Russell D. Petty

**Affiliations:** 1 Medical Oncology Group (Level 7, Corridor C), Division of Molecular and Clinical Medicine, Ninewells Hospital and School of Medicine, University of Dundee, James Arnott Drive, Dundee, DD1 9SY UK; 2 Medical Genetics, School of Medicine, Medical Sciences and Nutrition, University of Aberdeen, Aberdeen, AB25 2ZD UK; 3 Pathology Group, School of Medicine, Medical Sciences and Nutrition, University of Aberdeen, Foresterhill, Aberdeen, AB25 2ZD UK; 4 Department of Pathology, Ninewells Hospital and Medical School, Dundee, DD1 9SY UK

**Keywords:** Gastroesophageal cancer, Gefitinib, EGFR, Docetaxel, Platinum, ESCC, Chemotherapy combinations

## Abstract

**Purpose:**

Oesophageal squamous cell carcinoma (ESCC) has a poor prognosis. Advanced tumours are treated with fluoropyrimidine/platinum chemotherapy followed by irinotecan or taxane monotherapy, but resistance is common and new treatments are needed. Approximately 20% of ESCCs carry copy number gain (CNG) of the epidermal growth factor receptor (EGFR) gene. Previous trials show that while anti-EGFR monotherapy benefits biomarker-selected patients with EGFR CNG and/or high EGFR expression, combining anti-EGFR therapies with platinum fluoropyrimidine chemotherapies is not effective, and uncertainty remains regarding the optimal cytotoxic chemotherapy partner for anti-EGFR therapies in ESCC.

**Methods:**

The effects of *EGFR* CNG on fluoropyrimidine/platinum chemotherapy sensitivity in a cohort of gastroesophageal cancer patients (*n* = 302) was evaluated. Drug combination studies using the EGFR inhibitor gefitinib with cytotoxic chemotherapies, docetaxel, cisplatin, oxaliplatin and irinotecan, on cell proliferation and cell death of *EGFR* CNG ESCC cell lines were assessed.

**Results:**

EGFR CNG in gastroesophageal cancer patients was associated with improved overall survival following fluoropyrimidine/platinum chemotherapy. However, co-administration of gefitinib and oxaliplatin or cisplatin was frequently antagonistic in cell-based assays in EGFR CNG ESCC, whereas the combination of gefitinib with docetaxel or irinotecan was more efficacious. Co-administration of gefitinib/docetaxel and sequential administration of docetaxel before gefitinib showed synergy, but docetaxel given after gefitinib was antagonistic.

**Conclusion:**

Gefitinib/platinum co-administration demonstrated antagonism suggesting a possible explanation for the lack of benefit from addition of anti-EGFR therapies to fluoropyrimidine/platinum chemotherapy in trials. Gefitinib/docetaxel co-administration demonstrated synergy suggesting taxanes could be the most effective cytotoxic partner for anti-EGFR therapies in *EGFR* CNG-positive ESCC, but careful consideration of drug scheduling is required.

## Background

Oesophageal cancer is the sixth most common cause of death from cancer globally, and squamous cell carcinomas of the oesophagus (ESCC), is the dominant histological subtype of oesophageal cancer worldwide [1]. Patients frequently present with advanced disease and, as a result of late stage diagnosis and limited treatment options, 5-year survival rates remain low at around 15%. Current treatments depend on the tumour stage, co-morbidities and patient performance status; surgery is curative in fewer than half of patients and the majority of patients receive palliative treatment, including chemotherapy [2]. Currently, cytotoxic chemotherapy provides a systemic therapy option for palliative treatment of ESCC, but there are no licenced targeted therapies or predictive biomarkers and, therefore, an unmet need for more effective approaches [3].

First-line palliative chemotherapy usually involves a fluoropyrimidine/platinum combination but, eventually, all patients develop progressive disease with some receiving second-line treatment with a taxane or irinotecan monotherapy [4]. Recently, a study in patients progressing after fluoropyrimidine/platinum chemotherapy demonstrated that the PD-1 inhibitor nivolumab improved overall survival compared to taxane monotherapy (ATTRACTION-3 trial [5]). Although the progression free survival and the proportion of patients responding were similar in both groups, the responses to nivolumab were more durable but took longer to occur than responses to taxanes. These findings highlight the importance of identifying the minority subgroup of patients who would benefit long-term from nivolumab, but, in the short term, taxanes are superior. However, the low objective response rate (20%) and poor long-term survival with taxanes in this setting, indicates that treatment resistance is a major clinical challenge that needs to be addressed.

One approach to develop novel therapies is to identify and target oncogenic drivers and efforts to characterise genome alterations within tumour tissue is now enabling the selection of biomarkers for precision medicine targeted therapies. Potential drivers of oesophageal tumourigenesis include the epidermal growth factor receptor (EGFR). Copy number gain is detected in around 20% of tumours [6, 7], while EGFR is overexpressed in around 50% of ESCC tumours and correlates significantly with tumour invasion [6]. Targeting EGFR with EGFR tyrosine kinase inhibitors (TKi), such as gefitinib, erlotinib or afatinib, inhibits the proliferation of oesophageal cancer cell lines in vitro [8, 9], but, clinical trials of EGFR inhibitors in oesophageal cancer, including ESCC, have shown mixed results. Monotherapy trials in unselected patients with EGFR inhibitors indicate that there is an EGFR-driven minority ESCC subgroup who gain survival, symptomatic control and health-related quality of life benefits from EGFR inhibitors [10, 11]. In ESCC, *EGFR* CNG assessed by FISH, and/or EGFR protein over-expression have shown promise as predictive biomarkers to identify this benefiting subgroup, but needs prospective validation [3, 7, 12, 13]. Cell line models and patient-derived xenografts also demonstrate an EGFR-driven subgroup of ESCC sensitive to EGFR inhibitors and characterised by *EGFR* CNG and/or EGFR protein over-expression. However, even in these biomarker-selected groups, intrinsic and acquired resistance to EGFR inhibitors remains significant [8, 14, 15]. The considerable heterogeneity of *EGFR* CNG and protein over-expression observed in ESCC may be a key determinant of resistance [16], with rapid selection and outgrowth occurring of *EGFR* CNG and protein overexpression-negative tumour cell sub-clones that are unresponsive to EGFR inhibitors. This emphasises the importance of combining EGFR inhibitors with a therapy that is effective against EGFR ‘negative’ sub-clones, and ideally one that would also synergise with EGFR inhibitors towards the EGFR ‘positive’ driven sub-clones. To address this, a number of clinical trials have investigated the combination of EGFR inhibitors and cytotoxic chemotherapy. Clinical trials combining EGFR inhibitors and platinum/fluoropyrimidine chemotherapy in the advanced stage setting or with platinum fluoropyrimidine-based concurrent chemoradiotherapy in the curative treatment setting, have not shown an incremental benefit [17]. In the largest randomised trial in ESCC, in molecularly unselected patients with advanced stage disease, the addition of the anti-EGFR monoclonal antibody pantitumumab to cisplatin and 5FU chemotherapy did not improve overall survival [18]. Similarly, in unselected advanced stage gastroesophageal adenocarcinoma (GOA) patients, a negative impact on overall survival was observed with addition of panitumumab to epirubicin, oxaliplatin and capecitabine [19, 20]. Conflicting results have also been reported in trials of platinum-based chemotherapy in combination with EGFR TKi in non-small cell lung cancer patients (NSCLC) [21–23]. In contrast, the addition of the EGFR TKi erlotinib, to definitive chemoradiotherapy for ESCC, which included a taxane (paclitaxel and cisplatin) was beneficial [24].

Overall, there is evidence of an EGFR-driven and EGFR inhibitor-responsive subgroup of ESCC and, thus, the potential to combine current standard of care cytotoxic chemotherapies with EGFR inhibitors to improve outcomes. The development and clinical use of EGFR inhibitors for oesophageal cancer, however, has been hampered by conflicting trial results, which could be a consequence of biomarker-unselected patient cohorts, and also because questions regarding potential antagonistic effects of co-administration of EGFR inhibitors with cytotoxic chemotherapy have been raised [25–28]. Furthermore, the clinical relevance of this is increased by findings which suggest that, consistent with observation in other tumour types, EGFR signalling is a key determinant of resistance to immune checkpoint inhibitors in ESCC [29], and accordingly EGFR-driven ESCC are likely to be a subgroup that derive less, or no, benefit from immune checkpoint inhibitors.

To address this, and to identify the drug combinations most likely to benefit ESCC patients, in this study, we have investigated the outcomes from platinum/fluoropyrimidine chemotherapy in *EGFR* CNG positive and negative gastroesophageal cancer patients and then the combinatorial activity of EGFR inhibitors with cytotoxic drugs in ESCC cell lines with *EGFR* CNG and varying intrinsic sensitivity to gefitinib. Cytotoxic drugs included platinum-based chemotherapies (cisplatin and oxaliplatin), a taxane (docetaxel) and a topoisomerase inhibitor (irinotecan) which were tested in concurrent and sequential administration settings.

## Methods

### Patients and tumour samples

*EGFR* FISH was performed, to classify tumours as *EGFR* copy number gain (CNG) positive with either high polysomy (defined as having *EGFR* copy number ≥ 4 in ≥ 40% of cells) or gene amplification (defined by presence of tight gene clusters and a ratio of gene/chromosome per cell ≥ 2, or ≥ 15 copies of the genes per cell in ≥ 10% of analysed cells) or *EGFR* CNG negative (*EGFR* disomy, low trisomy, high trisomy and low polysomy) as described previously [16, 30], on formalin-fixed paraffin embedded tumour samples from the following patient cohorts (Table 1). First, a consecutive cohort of 52 patients with advanced stage (TNM version 7), gastroesophageal cancer treated with platinum/fluoropyrimidine-based palliative chemotherapy in 2015 at Tayside Cancer Centre (Table 1); all patients received up to 6 cycles of epirubicin 50 mg/m^2^ intravenously, day 1 cisplatin 60 mg/m^2^ or oxaliplatin 130 mg/m^2^ intravenously on day 1, plus capecitabine 1250 mg/m^2^ orally days 1–21 as 2 divided doses, or a continuous intravenous infusion of 5-fluorouracil 200 mg/m^2^/24 h, days 1–21 on a 21 day cycle. Secondly, a consecutive cohort of 250 patients with operable gastroesophageal cancer (TNM version 7) treated with surgical resection ± perioperative chemotherapy with 3 cycles before surgery and 3 cycles after surgery of: epirubicin 50 mg/m^2^ intravenously on day 1, cisplatin 60 mg/m^2^ intravenously on day 1, plus capecitabine 1250 mg/m^2^ orally days 1–21 as 2 divided doses, or a continuous intravenous infusion of 5-fluorouracil 200 mg/m^2^/24 h on days 1–21 on a 21-day cycle (Table 1) between 2004 and 2009 in Ninewells Hospital Dundee or Aberdeen Royal Infirmary. The use of all tumour specimens and clinical data was consistent with the patient consent provided and was approved by the appropriate UK regional research ethics committees prior to the work being undertaken.

**Table 1:** Clinical features of patients tested for epidermal growth factor receptor (EGFR) copy number gain (CNG)

(a) Neoadjuvant cohort			
Clinical feature	EGFR copy number gain (*N* = 40)	EGFR no copy number gain (*N* = 109)	*p*
Age, mean (SD)	64.3 (10.1)	64.9 (9.4)	0.733
Sex, no. (%)			
Male Female	25 (62.5%) 15 (37.5%)	72 (66%) 37 (37%)	0.687
Histological diagnosis, no. (%)			
Squamous Adenocarcinoma Other	8 (20%) 31 (77.5%) 1 (2.5%)	18 (16.6%) 83 (76.1%) 8 (7.3%)	0.51
Disease site, no. (%)			
Oesophageal Junctional Gastric	23 (57.5%) 1 (2.5%) 16 (40%)	53 (48.6%) 16 (14.7%) 40 (36.7%)	0.114
Stage, no. (%)			
I II III IV	7 (17.5%) 11 (27.5%) 20 (50%) 2 (5%)	19 (17.4%) 34 (31.2%) 53 (48.6%) 3 (2.8%)	0.90
Neoadjuvant chemotherapy, no. (%)			
Yes No	29 (72.5%) 11 (27.5%)	71 (65.1%) 38 (34.9%)	0.35

(b) Advanced stage cohort			
Clinical feature	EGFR copy number gain (*N* = 25)	EGFR no copy number gain (*N* = 27)	*p*
Age, mean (SD)	63.1 (9.6)	59.7 (8.8)	0.191
Sex, no. (%)			
Male Female	22 (88%) 3 (12%)	18 (67%) 9 (33%)	0.068
Histological diagnosis, no. (%)			
Squamous Adenocarcinoma Other	5 (20%) 19 (76%) 1 (4%)	6 (22.2%) 19 (70.3%) 2 (7.4%)	0.84
Disease site, no. (%)			
Oesophageal Junctional Gastric	19 (76%) 6 (24%) 0 (0%)	19 (70.3%) 8 (29.6%) 0 (0%)	0.647
Stage, no. (%)			
III IV	3 (12%) 22 (88%)	6 (22.2%) 21 (77.8%)	0.33

### Cell lines

Human KYSE520, OE21, and TE8 oesophageal squamous carcinoma cells (ESCC) with 14, 14, and 11 *EGFR* CNG, respectively, were obtained from the Cell Resource Center for Biomedical Research, Institute of Development, Aging and Cancer, Tohoku University, Japan. The cell lines were passaged in Roswell Park Memorial Institute RPMI medium 1640 supplemented with l-glutamine (GIBCO) and 10% foetal bovine serum (FBS) (GIBCO). Cells were tested negative for mycoplasma by the in-house testing facility (Mycoalert) and were authenticated by STR profiling (NorthGene Ltd, Newcastle UK).

### Reagents

Stock solutions were prepared as follows: Gefitinib (Iressa) (Tocris), 20 mM in DMSO; cisplatin [*cis*-Diamineplatinum (II) dichloride, (Sigma Adrich), 3 mM in sterile PBS; oxaliplatin (Selleckchem), 10 mM in sterile water; docetaxel (Selleckchem), 20 µM in DMSO and irinotecan (Tocris) (SN-38—active metabolite of CPT-11)], 20 mM in DMSO.

### Cell proliferation assays

1000 (OE21, KYSE520, and TE8) viable cells/well were seeded overnight in 96-well plates. Cells were then treated with either solvent control or two or fourfold dilutions of gefitinib, docetaxel, cisplatin, oxaliplatin or SN-38 (active metabolite of irinotecan). Where possible, drug titrations used were selected to be within the range of reported peak plasma concentrations of each drug which are: gefitinib—1–1.4 µM [31], oxaliplatin—3.6 µM [32], cisplatin—165 µM [33], docetaxel—4 µM [34], SN-38—0.03–0.17 µM [35]. The relative insolubility of cisplatin in PBS restricted its maximum working concentration (40 µM) to below peak plasma levels. Proliferation assay endpoints (control wells 80% confluent during log-phase growth) were analysed by CellTitre-Glo^®^ luminescent cell viability assay (Promega) according to the manufacturers’ instructions.

### Spheroid assays

Microtitre plates (TPP, U-bottomed) were coated with 0.5% poly(2-hydroxyethyl methacrylate) (poly-HEMA) prepared in 95% ethanol (two applications of 50 µL dried at 37 °C) and were stored at 4 °C until required. TE8 cells (1000 cells/100µL in growth media) were seeded into the poly-HEMA coated plates and were centrifuged for 10 min, 2000 rpm. After overnight incubation, tumour cell aggregates/spheroids were treated with 100 µL of the appropriate drug concentrations for 5 days before analysis by CellTitre-glo^®^ assay.

### Colony formation assay

OE21 cells were seeded overnight at 1000 cells/well in the middle wells of 24-well plates to avoid edge effects, before being treated with appropriate drug concentrations. Docetaxel and gefitinib monotherapy doses were selected to induce approximately 50% reduction in colony number. After seven days media was removed, colonies washed in PBS, and then fixed in methanol for 30 min. Plates were washed, air dried and colonies stained with crystal violet staining solution (0.5%w/v in 20% methanol). Excess stain was washed off with water, and the plates air-dried before scanning. Stained colonies were quantified by solubilisation in 1% SDS, and the absorbance of the resulting solution measured at 570 nm.

### Cell death assays

The proportion of dead cells was determined by CellTox™ Green cytotoxicity assay reagent (Promega) by imaging prior to, and following, addition of 50µL triton-x aqueous permeabilising solution (0.2%); non-viable cells were first labelled with CellTox™ Green cytotoxicity assay reagent (Promega) (4 µL/mL, 10 µL/well) and monitored during a drug treatment time course by IncuCyte^®^ Zoom real-time imaging and software (Essen Biosciences, Sartorius) (first reading). The percentage of dead cells was then determined by permeabilisation/dye-uptake and imaging of total cell number (second reading) after triton addition and the equation (first reading/second reading)*100.

### Drug co-administration

Studies were designed to conform to the requirements outlined for analysis by the Chou–Talalay mathematical model of drug combinations [36], namely, that combination drugs were used at equimolar dilution ratios at predetermined concentrations, where they had an effect on cell growth (around the IC_50_ values determined by prior CellTitre-Glo^®^ cell proliferation assays). Cells were seeded at 1000 cells/well in 96-well plates, divided into four groups and treated for four days as follows: solvent control group; gefitinib alone; cytotoxic drug alone; concurrent group (gefitinib plus cytotoxic). Cell proliferation was assessed by CellTitre-Glo^®^ assay.

### Sequential drug administration study design

Cells were seeded overnight in 96-well microtitre plates and were divided into six groups for treatment for 96-h as follows: (1) solvent control group, (2) cytotoxic drug alone group—cells were treated continuously with docetaxel, cisplatin or oxaliplatin, (3) gefitinib alone group—cells were treated continuously with gefitinib, (4) cytotoxic drug followed by gefitinib group—cells were incubated with docetaxel or cisplatin or oxaliplatin or 48 h followed by addition of gefitinib for 48 h, (5) gefitinib followed by cytotoxic group—cells were treated with gefitinib for 48 h followed by addition of docetaxel or cisplatin or oxaliplatin for 48 h. (6) Concurrent group (both)—cells were incubated concurrently with cytotoxic chemotherapy and gefitinib for 96 h. All groups were retreated with the appropriate drug dilution on each treatment day and drug dilutions in media were balanced for solvent concentration. No drug washouts were carried out. Cell proliferation was assessed by CellTitre-Glo^®^ assay.

### Statistical analysis

Survival analysis was performed using IBM SPSS statistics v22 (IBM Corporation, Armonk, NY, USA). Kaplan–Meier and Cox proportional hazards analysis were used for survival analysis and survival time was calculated in days from the date of histological diagnosis until the date of death. All reported *p* values are two sided. A *p* value of < 0.05 was considered statistically significant. ANOVA was performed using Graphpad prism software.

IC_50_ values were determined from cell proliferation assays using CalcuSyn (Biosoft Version 2.0) or Graphpad prism software. The antiproliferative effect of combination treatments was evaluated by determining the drug combination index (CI). Results were analysed according to the Chou–Talalay method [36] using CalcuSyn software (CalcuSyn, Inc. Paramus, USA) which generates Dm values (IC50), dose response curves and median effect plots. Recommended symbols for describing synergistic, additive or antagonistic effects in drug combination studies analysed with the CI method (CalcuSyn user manual) are given where appropriate.

## Results

### *EGFR* CNG status and outcomes from Platinum/fluoropyrimidine chemotherapy in gastroesophageal cancer patients.

First, we investigated the impact of EGFR signalling on clinical chemosensitivity by analysing outcomes in *EGFR* CNG positive and negative gastroesophageal cancer patients treated with platinum–fluoropyrimidine combination chemotherapy (PBC).

**Fig. 1:**
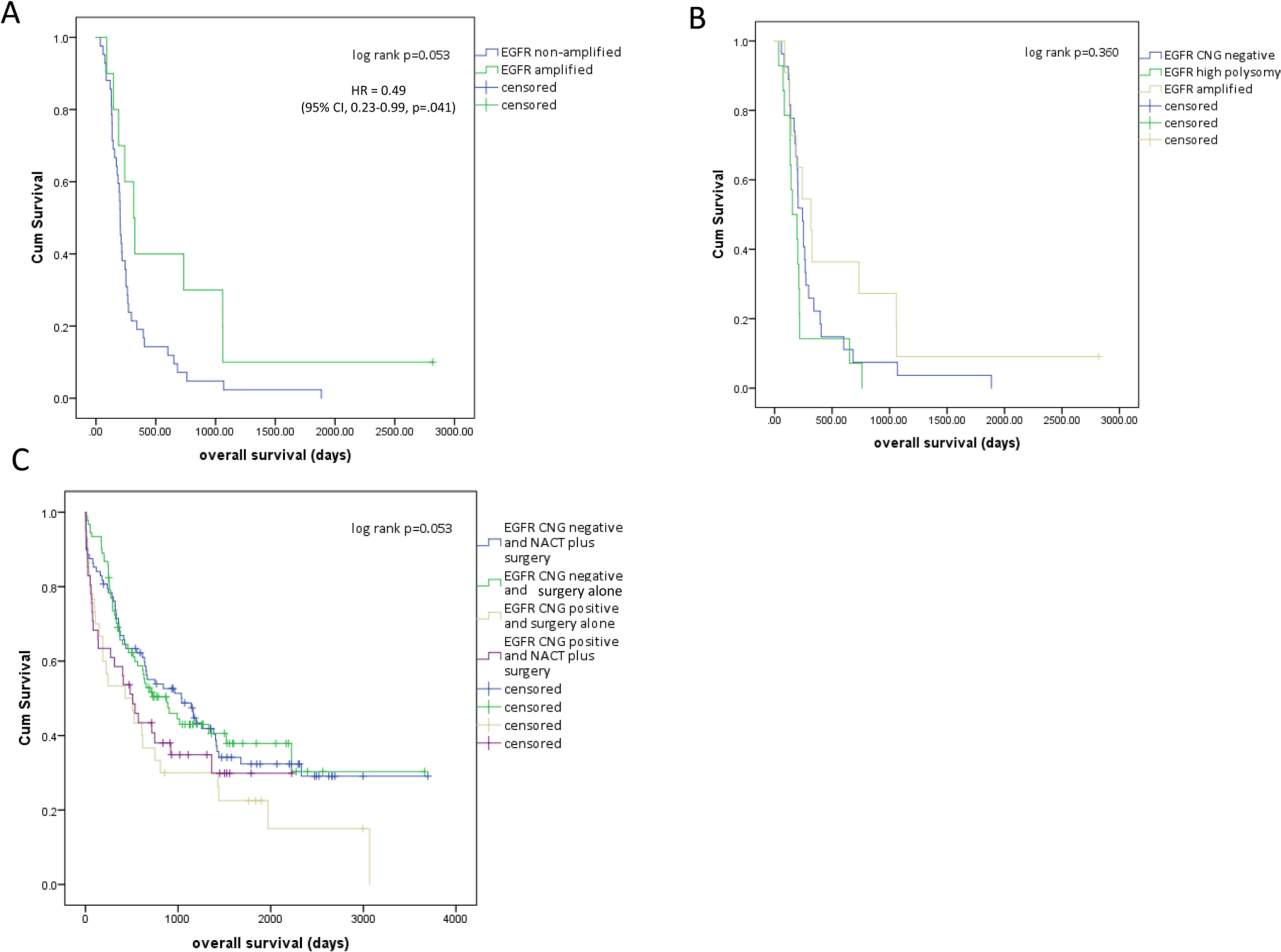
*EGFR* copy number status and outcome following treatment with platinum-based chemotherapy in gastroesophageal cancers. **a** Advanced stage patients (*n* = 52) treated with palliative platinum-based combination chemotherapy categorised as *EGFR* amplified and *EGFR* non-amplified. **b** Advanced stage patients treated with palliative platinum-based combination chemotherapy categorised as *EGFR* amplified, *EGFR* high polysomy or *EGFR* copy number gain negative (CNG negative includes *EGFR* disomy, low trisomy, high trisomy and low polysomy). **c** Operable gastroesophageal cancers treated with surgical resection alone or platinum-based neo-adjuvant chemotherapy (NACT) followed by surgical resection categorised as *EGFR* copy number gain positive (CNG positive, includes *EGFR* amplification and high polysomy), or *EGFR* CNG negative

*EGFR* CNG status was not associated with patient clinical features (Table 1). Analysis of the cohort (*n* = 52) of advanced gastroesophageal cancer patients treated with palliative PBC revealed that patients with tumours containing amplified *EGFR* (*n* = 13) had longer median survival (315 days, 95% CI 183.3–446.7) than patients without *EGFR* CNG (201 days, 95% CI 184.1–217.9), HR 0.49, 95% CI 0.23–0.99, *p* = 0.041 (Fig. 1a). Shorter survival times compared to amplified *EGFR* cases, were also noted in patients with high polysomy (defined as having *EGFR* copy number ≥ 4 in ≥ 40% of cells) [16] (Fig. 1b).

Operable patients with *EGFR* CNG positive tumours (high polysomy or amplification) had shorter median overall survival (284 days, 95% CI 284.5–737.5) than patients with *EGFR* CNG negative tumours (905 days 95% CI 566.9–1243.1), HR 1.51, 95% CI 1.09–2.12, *p* = 0.016). However, when analysed according to treatment received in this cohort, patients with *EGFR* CNG positive tumours who received neoadjuvant PBC had longer overall survival than patients with *EGFR* CNG positive tumours who did not receive neoadjuvant PBC (Fig. 1c). Patients without *EGFR* CNG positive tumours had similar overall survival regardless of whether they received pre-operative PBC or not (Fig. 1c). *EGFR* CNG positive patients who received neoadjuvant PBC had similar overall survival to those without *EGFR* CNG, but *EGFR* CNG positive patients who did not receive neoadjuvant PBC had shorter overall survival (Fig. 1c).

Overall, this suggests that gastroesophageal cancer patients with EGFR-driven tumours (as identified by *EGFR* CNG) benefit from, and are more sensitive to, PBC. This implies that therapeutic inhibition of EGFR-oncogenic pathways in *EGFR* CNG positive patients could negatively impact on the expected benefit derived from platinum-based chemotherapy and would be antagonistic. These observations could provide an explanation for negative clinical trials investigating PBC combined with EGFR inhibitors in gastroesophageal cancer.

### Combinations of gefitinib and cytotoxic chemotherapy in *EGFR* CNG ESCC cell lines

**Fig. 2:**
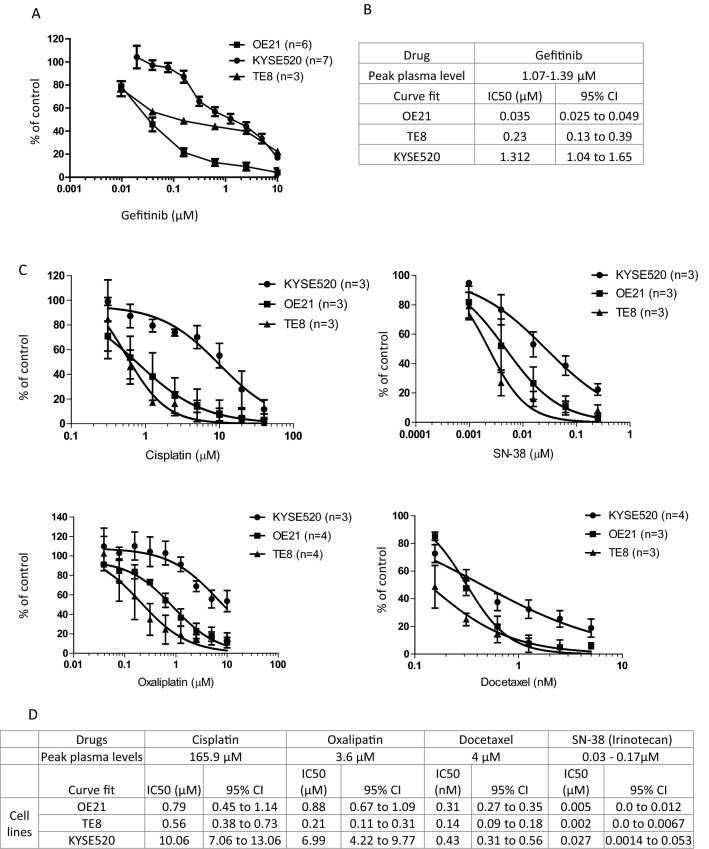
Sensitivity of ESCC cell lines to gefitinib and cytotoxic monotherapy. Dose–response curves (**a**, **c**) and IC50s (**b**, **d**) for gefitinib (**a**, **b**) and cytotoxic agents, cisplatin, oxaliplatin, docetaxel, and irinotecan (**c**, **d**) in three EGFR dysregulated ESCC cell lines. **a**, **c** Cells were seeded overnight in 96-well plates and treated with concentrations of gefitinib, docetaxel, cisplatin, oxaliplatin or SN38 (irinotecan) as indicated. Cells were harvested with CellTitre-Glo^®^ assay reagent (Promega) and graphs depict cell proliferation relative to solvent control treated cells (set at 100%). The non-linear curve fit was generated using Graphpad from at least three independent assays (*n*) as indicated in the graph legend. IC50 values and 95% confidence intervals (**b**, **d**) were determined using graphpad prism. Peak plasma levels are also given for each agent

No gastroesophageal adenocarcinoma cell lines with *EGFR* CNG are available, so to investigate the potential antagonistic interaction between EGFR inhibitors and oxaliplatin and cisplatin, our subsequent experiments focused on ESCC. Three ESCC cell lines with *EGFR* copy number gain were selected. KYSE520 cells were considered resistant to gefitinib, having an IC50 at around the peak plasma levels (Fig. 2a), while inhibition of proliferation of OE21 and TE8 cells occurred at IC50s of 30-fold and fivefold lower than peak plasma levels, respectively (Fig. 2b). This range in sensitivity to gefitinib reflected the range in response seen in patients in the clinical setting. We also determined the sensitivity of these cell lines to oxaliplatin, cisplatin, docetaxel and irinotecan (administered as the active metabolite SN38) (Fig. 2c, d). KYSE520 cells were also least sensitive to cytotoxic agents in agreement with genomics of drug sensitivity in cancer data (https://www.cancerrxgene.org), while TE8 and OE21 cells were relatively sensitive (Fig. 2d).

**Fig. 3:**
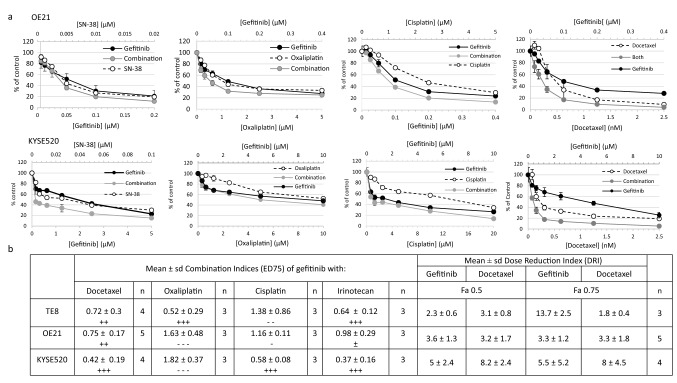
Dose response curves of gefitinib co-administration with cytotoxic chemotherapies. **a** Gefitinib sensitive (OE21) and gefitinib resistant (KYSE520) cells were treated with gefitinib alone, cytotoxic chemotherapy alone or equimolar titrations of both drugs (combination). Cell proliferation was determined by CellTitre-Glo^®^ assay, and representative graphs depict proliferation relative to solvent control treated cells (set at 100%). **b** Table of combination indices (CI) and dose reduction indices (DRI) of gefitinib and cytotoxic chemotherapies in ESCC cell lines. Average (± SD) CI values at ED75 determined by CalcuSyn software from at least three (*n*) independent assays [representative dose response curves are shown in **a**]. CI values < 1 indicate synergistic interactions, CI = 1 additive, CI values > 1 indicate antagonistic interactions. CalcuSyn recommended symbols are also provided to indicate the degree of the effect: antagonistic (– – –); moderate antagonism (– –); slight antagonism (−); nearly additive (±); moderate synergism (++); synergism (+++). Mean (± SD) DRI determined from independent 96-h assays (*n*) of gefitinib in combination with docetaxel evaluated by endpoint CellTitre-Glo^®^. DRI were calculated by CalcuSyn software. Fa = fraction affected equivalent to ED50 and ED75 at 0.5 and 0.75, respectively

Having determined the dose response of the agents, we conducted combination experiments in gefitinib sensitive (OE21, TE8) and resistant (KYSE520) cells using drug titrations at equimolar ratios (representative dose responses in OE21 and KYSE520 cells are shown in Fig. 3a) and then calculated combination indices using CalcuSyn software based on Chou–Talalay methodology. Mean ED75 combination indices ± SD from independent experiments are summarised in Fig. 3b. Consistent with our hypothesis, platinum-based cytotoxic drugs, cisplatin and oxaliplatin, frequently had antagonistic activity when used in combination with gefitinib (CI > 1). The level of antagonism varied the with agent and cell line. Both cisplatin and oxaliplatin were antagonistic in combination with gefitinib in OE21 cells, the line most sensitive to gefitinib as a monotherapy. SN38 combined with gefitinib induced responses ranging from nearly additive to synergistic. This observation is consistent with reports of synergistic interactions between irinotecan and gefitinib in colorectal cancer cell lines [37]. Docetaxel plus gefitinib, however, consistently showed synergistic activity across the cell line panel, and was highly effective at inhibiting the proliferation of the previously gefitinib refractory cell line KYSE520. Importantly, the combination of gefitinib with docetaxel led to dose reduction indices (DRI) for gefitinib (ED50, range 2.3–5 fold; ED75 range 3.3–13.7 fold) and docetaxel (ED50, range 3.1–8.2 fold; ED75 range 1.8–8 fold) across the cell line panel (Fig. 3b). These data suggest that similar efficacy can be achieved using reduced doses of gefitinib and docetaxel when used in combination.

**Fig. 4:**
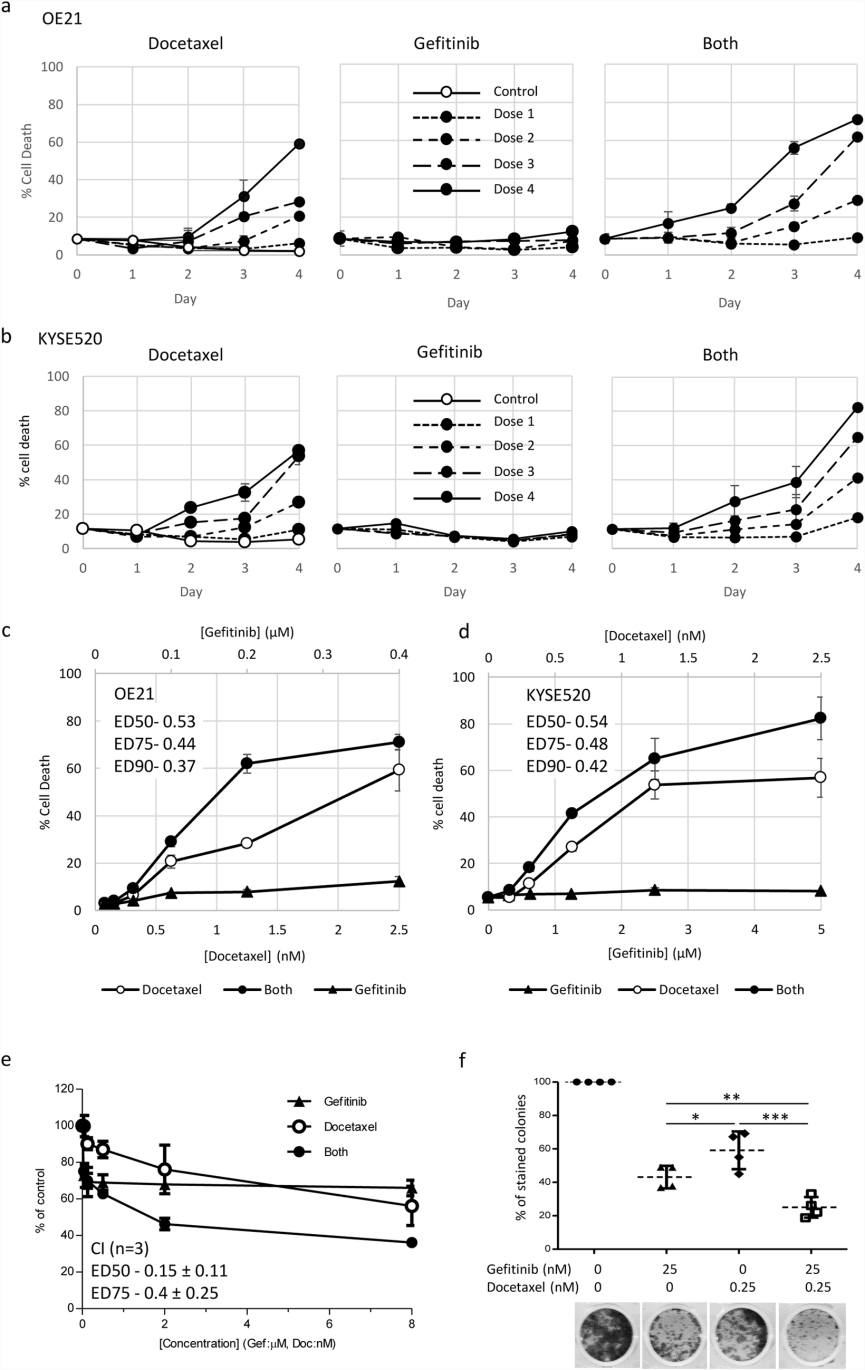
Docetaxel in combination with gefitinib synergistically enhances ESCC cell death. Cytotoxicity of drug treatments (docetaxel alone, gefitinib alone or gefitinib plus docetaxel combination) on the ESCC cell lines OE21 (**a**, **c**) and KYSE520 (**b**, **d**) was assessed by CellTox™ green assay and imaging (IncuCyte^®^ Zoom). **a**, **b** Time course of treatment: cells were treated for 4 days with solvent (control) increasing doses of docetaxel (OE21 and KYSE520: dose 1 = 0.3215 nM; dose 2 = 0.625 nM; dose 3 = 1.25 nM; dose 4 = 2.5 nM), gefitinib (sensitive line OE21: dose 1 = 0.05 µM; dose 2 = 0.1 µM; dose 3 = 0.2 µM;, dose 4 = 0.4 µM and resistant line KYSE520: dose 1 = 1.25 µM; dose 2 = 2.5 µM; dose 3 = 5 µM;, dose 4 = 10 µM) or both drugs combined (both). **c**, **d** Endpoint IncuCyte^®^ data from day 4 are presented as dose response curves in OE21 (**c**) and KYSE520 (**d**) (mean ± SEM of at least three replicate wells, 4 fields per well of a representative assay). Synergistic combination indices at effective dose 50%, 75% and 90% (ED50, ED75 and ED90) are indicated. **e** Five-day TE8 3-D anchorage independent spheroid assays treated with equimolar fourfold dilution series of gefitinib (from 8 µM) and docetaxel (from 8 nM) as single agents or in combination. Synergy ED50 and ED75 CI values were calculated using CalcuSyn and are presented as the mean CI ± SD from three independent assays. (ED90s were not reached and calculated CI ED90 were variable across independent assays). **f** OE21 colony formation assay using docetaxel and gefitinib doses selected to induce approximately 50% reduction in colony number as a monotherapy, and in combination. Statistical comparisons were performed using Graphpad prism software (ANOVA plus Bonferroni correction for multiple testing from *n* = 4 independent assays; *p = 0.01–0.05, **p = 0.001–0.01, ***p =  < 0.001)

We next tested docetaxel and gefitinib as monotherapy and combination therapy in kinetic cell death assays in OE21 and KYSE520 cells over increasing doses (dose 1–4) (Fig. 4). As expected, gefitinib induced little cell death over the 4-day time course in line with its primary mode of action being induction of G_1_ cell cycle arrest. Docetaxel induced dose-dependent increases in cell death in both cell lines. When docetaxel and gefitinib were used in combination, synergistic levels of cell death were induced (Fig. 4c, d) and with more rapid kinetics (Fig. 4a). In addition, we tested responses to gefitinib and docetaxel in 3-D cell aggregates/spheroids and 2D colony formation, which can more closely model aspects of in vivo tumour biology such as reduced proliferation rates with increased survival, cell: cell adhesions, hypoxic cores and long term replicative capacity (colony formation). Gefitinib and docetaxel displayed synergistic activity against TE8 cell spheroids at ED50 and ED75 (Fig. 4e) (CI ED50, 0.15 ± 0.11; ED75, 0.4 ± 0.25. *n* = 3). OE21 and KYSE520 cells did not survive for the duration of the 5-day spheroid assay so we were unable to use these cells; however, OE21 cells were able to form discrete 2-D colonies in colony formation assays and OE21 colonies were more significantly inhibited by the combination of gefitinib and docetaxel than either drug as a monotherapy (*n* = 4). These results indicated that gefitinib in combination with docetaxel had the most consistent activity in ESCC inducing synergistic effects on proliferation and cell death in 2-D and 3-D model systems.

**Fig. 5:**
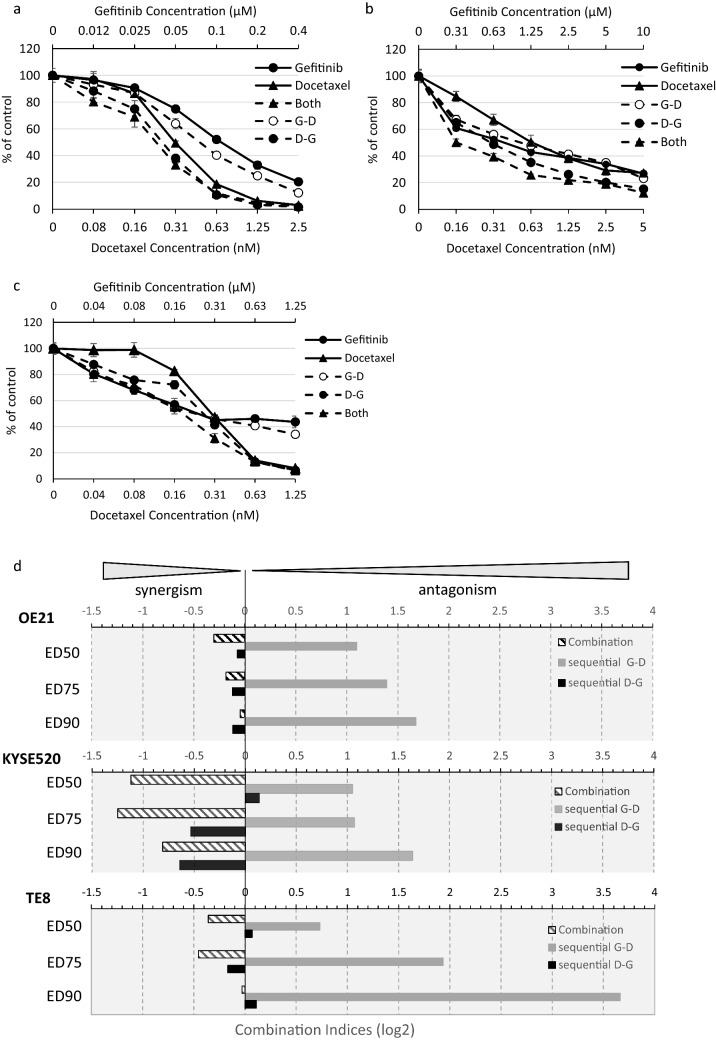
Efficacy of docetaxel/gefitinib combination treatment is sequence-dependent. **a**–**c** Representative dose response curves of gefitinib sensitive OE21 (**a**), gefitinib resistant KYSE520 (**b**) and gefitinib sensitive TE8 (**c**) ESCC cells were treated for 4 days with gefitinib or docetaxel and both drugs either in combination or sequentially: 4 days both drugs (both), 48-h gefitinib treatment followed by addition of docetaxel for the remaining 48-h (G—D) or 48-h docetaxel treatment followed by addition of gefitinib for the remaining 48 h (D—G). Cells were analysed by CellTitre-Glo^®^ assay (**d**) Log_2_ combination indices were determined from assays described in **a**–c using CalcuSyn software, where 0 = additive effects, < 0 = synergistic effects, > 0 = antagonistic. log_2_ CI values at 50%, 75% and 90% effective dose ED50, ED75 and ED90, respectively, are displayed

To determine whether synergistic effects could be affected by dosing schedules we tested gefitinib and docetaxel in sequential treatments over 96 h, D—G = docetaxel followed by gefitinib; G—D = gefitinib followed by docetaxel and compared the effects on cell proliferation of concurrent (combination) gefitinib and docetaxel treatment (Fig. 5). As confirmation of our previous results, concurrent administration of gefitinib and docetaxel induced synergistic inhibition of proliferation of all three cell lines. Similar results were noted when docetaxel was given prior to gefitinib (schedule D—G). However, there was a striking shift in response when gefitinib was given prior to docetaxel (G—D). Administered sequentially, gefitinib followed by docetaxel was antagonistic in all three cell lines, suggesting that careful dosing schedules should be devised to avoid deleterious drug interactions.

## Discussion

Oesophageal squamous cell carcinoma (ESCC) patients whose tumours have *EGFR* CNG and/or EGFR protein overexpression may represent a subgroup that benefits from EGFR inhibitor monotherapy [7, 12, 13]. However, even in this biomarker-selected subgroup of ESCC, significant proportions of patients do not respond to EGFR inhibitors and durable responses are uncommon, indicating that primary and acquired clinical resistance is a major clinical challenge. Heterogeneity is a predominant feature of ESCC, including for *EGFR* CNG and protein over-expression. Tumours with a higher number of genomic clonal subpopulations that are not EGFR-driven are less likely to respond significantly to monotherapy with an EGFR inhibitor. Therefore, EGFR combination treatments are likely to be important to optimise treatment effectiveness in *EGFR* CNG positive ESCC.

Several clinical trials have investigated the combination of EGFR inhibitors with cytotoxic chemotherapy or concurrent chemoradiotherapy [17–20, 24]. In the palliative setting, in both ESCC and GOA, the addition of EGFR inhibitors to platinum plus fluoropyrimidine chemotherapy has not improved overall survival [18–20]. Similarly, in the radical treatment setting, the addition of EGFR inhibitors to concurrent chemoradiotherapy, with a platinum and fluoropyrimidine chemotherapy backbone has not improved overall survival [17]. However, addition of EGFR inhibition to chemoradiotherapy in ESCC with a chemotherapy backbone incorporating paclitaxel did improve overall survival, even in biomarker-unselected patients [24]. Consistent with these clinical trial results we observed that *EGFR* CNG positive gastroesophageal cancers in both the palliative and neoadjuvant setting appear to be more sensitive to platinum fluoropyrimidine-based cytotoxic chemotherapy. This suggests that the use of an EGFR inhibitor could reduce, or negate, the benefit of platinum-based cytotoxic chemotherapy in patients with EGFR-driven tumours and would thus be antagonistic. We confirmed drug antagonism in *EGFR* CNG cell lines. Because no GOA cell lines with *EGFR* CNG are available, our cell line experiments were restricted to ESCC. This is a limitation of our work, and deriving *EGFR* CNG GOA cell lines to confirm our findings would be advantageous. The finding of synergy between docetaxel and gefitinib observed in 2-D cell culture was supported by our findings in longer-term 2-D colony formation assays and in 3-D spheroid culture models. However, not all cell lines used in this study were able to grow in 3-D culture and likewise, to overcome this limitation of the current study, testing of additional *EGFR* CNG positive cell lines that are able to survive anchorage-independent seeding would be advantageous. Investigation of drug synergy in an in vivo xenograft model, evaluation of the impact of *EGFR* CNG on survival in a cohort of ESCC patients treated with docetaxel monotherapy, as well as extension of investigation to other EGFR inhibitors would also be beneficial. In addition, elucidation of the mechanism of synergy between docetaxel and gefitinib, or other EGFR inhibitors, in *EGFR* CNG positive ESCC would also provide additional understanding and may suggest predictive biomarkers to identify those patients that might benefit most from this combination.

The majority of earlier reports have suggested that EGFR protein expression and/or EGFR gene copy number gain are associated with shorter survival of gastroesophageal cancers including adenocarcinomas and ESCCs [38–40]. However, longer survival, or no impact, has been observed in other studies [38–40]. In contrast to our investigation, the influence of treatment modality in general and cytotoxic chemotherapy in particular on how EGFR impacts the clinical outcomes has not been investigated in the majority of these studies. Our data shows an interaction between neo-adjuvant PBC and the impact of EGFR and, consistent with the findings of previous studies, indicates that the treatment patients receive should be taken into account when the impact of EGFR is being considered. Furthermore, Smyth et al., reported that higher tumour EGFR RNA expression is associated with shorter overall survival in gastroesophageal cancer patients treated with peri-operative PBC [41]. These findings contrast with ours, and suggest that EGFR copy number measured by FISH and EGFR RNA expression measured by nanostring may be measuring different aspects of gastroesophageal cancer biology. For example EGFR FISH-positive tumours almost invariably overexpress EGFR RNA (and EGFR protein), but up to 50% of *EGFR* FISH-negative tumours also have high EGFR RNA and protein [42, 43]. The lack of benefit from gefitinib in *EGFR* CNG-negative patients (which might have still high expression EGFR RNA and or protein) suggests that EGFR protein or RNA expression may be a less reliable marker of EGFR-driven gastroesophageal cancers and a less reliable marker of the impact of EGFR signalling and EGFR inhibitors on PBC sensitivity, resistance or clinical outcomes. Nevertheless, these findings also indicate the importance of considering the type of EGFR assay used in gastroesophageal predictive and prognostic studies. To our knowledge the impact of *EGFR* CNG on overall survival in advanced stage patients treated with palliative platinum fluoropyrimidine chemotherapy (PBC) has not been previously reported.

Our data indicating that the combination of gefitinib with cisplatin is antagonistic (in TE8 and OE21 cells) are at odds with reports that treatment of TE8 xenograft tumours with cisplatin in combination with EGFR inhibition by cetuximab significantly reduces their size [44]. Such discrepancies may arise due to the nature of the mechanism of inhibition of EGFR (small molecule EGFR TKi versus blocking antibody with potential antibody-dependent cellular cytotoxicity effects). However, clinical results of EGFR monoclonal antibodies both as monotherapy and in combination with PBC in ESCC have been similar to those demonstrated with EGFR TKis [10, 18, 20–23, 27, 28, 35]. In addition, in line with our conclusions, the POWER phase III RCT in advanced ESCC did not demonstrate any benefit of the addition of the humanised monoclonal anti-EGFR antibody panitumumab to cisplatin plus fluoropyrimidine chemotherapy [18]. POWER enrolled molecularly unselected ESCC patients, but, a retrospective analysis demonstrated that EGFR IHC did not correlate significantly with overall survival, and *EGFR* copy number was not investigated. The antagonism between cisplatin and oxaliplatin and EGFR inhibition provides a key explanation for this, and other, negative clinical trials and suggests that, even if these trials had been undertaken in biomarker-selected patients, benefit from the addition of EGFR inhibitors may not have been observed.

Previous studies in KYSE30 cells have suggested that the sequence of administration of gefitinib in combination with cytotoxic agents determines efficacy. Synergy was noted with cisplatin, carboplatin, oxaliplatin, docetaxel and paclitaxel followed by gefitinib [45]. However, our studies in a wider cell panel suggest that the effect of concurrent gefitinib with platinum-based cytotoxic drugs is cell line-dependent and thus the effects of this combination may be unpredictable in the clinical setting. In contrast, we demonstrated that gefitinib and docetaxel was consistently synergistic. This observation is consistent with the demonstration that addition of erlotinib to chemoradiotherapy including paclitaxel was beneficial in ESCC [24]. In this study there was no biomarker selection, and we hypothesise that in this trial the greatest benefit from addition of erlotinib will be seen in those patients who are *EGFR* CNG and/or have EGFR protein overexpression. Our dose reduction analysis indicates that equivalent reductions in tumour cell growth could be achieved with lower doses of gefitinib and docetaxel if used in combination, which could have significant advantages in terms of reduced financial costs of the treatment and, more critically, reduced toxicity to the patient. The cell line studies also suggest that the sequence of administration of taxane and EGFR inhibitor is critical. The administration of gefitinib prior to docetaxel invariably resulted in antagonism which is consistent with studies in both an NSCLC cell line [46] and in KYSE30 ESCC cells [45]. This data suggests that careful scheduling, or drug holidays may be required to avoid possible antagonistic drug interactions. In support of this, when used in combination with paclitaxel, pulsatile administration of gefitinib has proved more effective than continuous dosing in murine models of breast cancer, [47].

The molecular mechanism of drug combination antagonism or synergy in this setting is unclear and is under investigation. In vitro studies analysing potential combination therapies on both head and neck SCC and NSCLC cell lines suggest that there is also antagonism between gefitinib and cisplatin in other tumour types [25–27]; the effects have been variously attributed to cisplatin cytotoxicity being dependent on EGFR phosphorylation and degradation [28], induction of epithelial to mesenchymal transition (EMT) which is associated with increased resistance to gefitinib [26], reduced cisplatin entry into the cell and increased DNA repair or cell cycle arrest in G_1_. However, antagonism can be overcome by concurrent use of autophagy inhibitors (without any apparent effect on the cell cycle) [27] suggesting that factors other than the phase of the cell cycle may be involved. Previous reports in colorectal cancer cells have suggested that treatment of cells with cytotoxic agents increases phosphorylation of EGFR rendering cells more sensitive to the effects of EGFR TKis, whereas antagonistic interactions result from a cytotoxic drug induced a decrease in EGFR phosphorylation [37, 48]. In NSCLC, the mechanism of synergistic interaction was also suggested to be due to increased docetaxel-induced phosphorylation of EGFR and its subsequent inhibition following gefitinib addition [6]; however, similar analysis of our ESCC cell lines over several repeat experiments was inconclusive. The antagonistic effect of sequential administration of gefitinib prior to docetaxel could be due to cell cycle effects, where gefitinib induces a G1 cell cycle arrest thus rendering taxanes (which are primarily mitotic spindle inhibitors acting in G2/M) ineffective [45, 46]. Since concurrent administration of gefitinib or administration after docetaxel is synergistic, in the clinic, this would suggest that an interrupted schedule of an oral TKi like gefitinib, might be needed in combination with a taxane. Alternatively, and in contrast to common clinical practice at present, an anti-EGFR monoclonal antibody should potentially be administered after docetaxel and not before, when combined with a taxane.

Recently, the ATTRACTION-3 study demonstrated that the PD-1 inhibitor nivolumab provided improved overall survival compared to taxane monotherapy suggesting that nivolumab is a new standard of care for ESCC after progression with fluoropyrimidine/platinum chemotherapy [5]. However, given that only a minority subgroup of patients respond to nivolumab and responses took a median of 2.6 months to occur, its use presents clinical challenges in this setting, where patients often have high tumour burdens and are very symptomatic. In addition, in squamous cell carcinomas including ESCC, EGFR activation is associated with depleted tumour infiltrating lymphocytes and resistance to immune checkpoint inhibition (ICI) [29]. EGFR activation leads to increased anaerobic glycolysis in tumour cells, glucose depletion and accumulation of lactate in squamous cell carcinomas, meaning that tumour-infiltrating T cells, may have to compete for metabolic fuels. ICI appear to be less effective in EGFR mutant positive NSCLC [29, 49], and EGFR activation has been associated with hyper-progression following ICI therapy [50]. Early phase trials in NSCLC have revealed problematic toxicity combining EGFR inhibitors and immune checkpoint inhibitors [51]. Together these data suggest that EGFR-driven ESCC, identified by *EGFR* CNG and /or EGFR protein overexpression are likely to be less sensitive to nivolumab which is unlikely as a monotherapy to provide an effective treatment for this group of patients. As such, taxanes will likely remain one standard of care for ESCC after progression with fluoropyrimidine/platinum chemotherapy, either before or after nivolumab. Overall, our results contribute additional evidence to support investigation of EGFR inhibitors in *EGFR* CNG positive ESCC and suggest that a combination strategy with taxanes has the potential for synergism thereby optimising clinical impact and effectiveness. Since taxanes are a standard of care for ESCC after progression with fluoropyrimidine/platinum chemotherapy evaluating the benefit of the addition of an EGFR inhibitor to docetaxel or paclitaxel in tumours that are *EGFR* CNG and/or EGFR protein overexpressed by IHC would be the most appropriate initial area of clinical investigation.

In summary, drug combination studies indicate that targeting EGFR in ESCC cells carrying *EGFR* copy number gain may negate or reduce anticancer effects of platinum-based chemotherapy; however, EGFR inhibitors are efficacious and synergistic in combination with docetaxel when scheduled correctly. We recommend further preclinical studies and consideration of clinical investigation of scheduled anti-EGFR therapies combined with taxanes for ESCC patients with tumours expressing high EGFR by IHC and/or *EGFR* CNG.

## Abbreviations

ESCC: Oesophageal squamous cell carcinoma
EGFR: Epidermal growth factor receptor
CNG: Copy number gain
EAC: Oesophageal adenocarcinoma
NSCLC: Non-small cell lung cancer
IHC: Immunohistochemistry
SD: Standard deviation
ICI: Immune checkpoint inhibition
TKi: Tyrosine kinase inhibitor
EMT: Epithelial to mesenchymal transition
GOA: Gastroesophageal adenocarcinoma
CI: Combination index
PBC: Platinum–fluoropyrimidine combination chemotherapy
